# Tailoring of the ongoing water, sanitation and hygiene interventions for prevention and control of COVID-19

**DOI:** 10.1186/s41182-020-00236-5

**Published:** 2020-06-16

**Authors:** Vivian Mushi, Magdalena Shao

**Affiliations:** 1 Department of Parasitology and Medical Entomology, School of Public Health and Social Sciences, Muhimbili University of Health and Allied Sciences, P.O. Box 65001, Dar es Salaam, Tanzania; 2 Department of Environmental and Occupational Health, School of Public Health and Social Sciences, Muhimbili University of Health and Allied Sciences, P.O. Box 65001, Dar es Salaam, Tanzania

**Keywords:** COVID-19, WASH, HWWS, Interventions and challenges

## Abstract

Water, sanitation, and hygiene (WASH) interventions remain to be important in the prevention of further spread of coronavirus disease-2019 (COVID-19). Basic hygiene interventions such as handwashing with water and soap (HWWS) when applied consistently will deactivate and remove the virus particles from the hands. Realizing the efforts that have been made by countries world over in controlling the COVID-19, this letter seeks to discuss how the available WASH services can be used in the fight against further spread of COVID-19. The letter highlights the challenges being faced by the current WASH services in middle- and low-income countries and suggests measures that can be employed to strengthen the WASH services in this period of the COVID-19 pandemic.

To the Editor,

Coronavirus disease (COVID-19) is an infectious disease caused by a novel coronavirus known as severe acute respiratory syndrome coronavirus 2 (SARS-CoV-2) [1]. It was December 31, 2019, when the outbreak of coronavirus disease was announced by the WHO country office in China [2]. On January 30, 2020, COVID-19 was declared a public health emergency of international concern, and within less than 3 months, the virus had spread in the whole world [2]. Up to now, the coronavirus has already spread in 217 countries and territories with more than 6,702,767 cases and at least 393,212 deaths worldwide have been reported [3].

The disease has no treatment or vaccine; however, several clinical trials are going on in different countries with the aim of testing some promising drugs [4]. While waiting for treatment and vaccine, we consider that preventive measures are the key to prevent further transmission of the disease. The World Health Organization (WHO) recommended public health measures such as regularly and thoroughly handwashing with water and soap or with alcohol sanitizers, covering of the mouth and nose with a mask in crowded areas, physical distancing for at least 6 feet from one person to another, adhering to good respiratory hygiene practices especially when sneezing or coughing by covering the nose and mouth with a bent elbow or tissue, and avoid visiting crowded places [5]. Apart from the measures that have been recommended by the WHO, some countries have initiated specific strategies to curb the COVID-19 outbreak such as partial or full lockdown for example in Europe (France, UK, Italy), North America (USA), Africa (South Africa), Asia Pacific (New Zealand), South Asia (India), Middle East (Saudi Arabia), and East Asia (China); restriction of visitors from affected countries; stopping of flights; mandatory 14 days quarantine for all arrivals from COVID-19-affected countries; and isolation of suspected persons [6, 7].

COVID-19 can be transmitted directly from person-to-person via respiratory droplets or contact routes. Contact transmission happens when a person with contaminated hands touch the eyes, nose, or mouth. Indirect contact transmission can occur when the virus is transferred from one surface to another by contaminated hands [8, 9]. Water, sanitation, and hygiene (WASH) practices are crucial in preventing human to human transmission of COVID-19 [10]. Currently, however, no studies have been conducted on the survival of SARS-CoV-2 in drinking water and sewage system; thus, we think that this is an opportune time to start thinking about the possibility of the virus to survive in these environments [10]. It has been documented with evidence that coronavirus was extracted in feces and viral RNA fragments have been recovered in fecal samples of the patients throughout their illness and after recovery [11]. In the meantime, hygienic measures particularly hand hygiene practices are essential to prevent further spread of COVID-19. Hand hygiene practices are imperative to curb transmission of ongoing infectious diseases caused by viruses, parasites, and bacteria [8].

However, it should be noted that handwashing with water and soap (HWWS) is not a new intervention especially in countries where bacterial infections, viral infections, and neglected tropical diseases are endemic. This intervention has been used as one of the silent weapons in WASH interventions to mitigate transmission of the diseases, and it has been proven to be effective in reducing transmission and disease risk especially during the infectious outbreak [12]. Furthermore, HWWS has shown to be a feasible and cost-effective mechanism to prevent infectious outbreaks [12]. Some studies have shown that HWWS can decrease the level of microorganisms close to zero and can also reduce the risk of respiratory infections by 23% [13, 14]. The existing WASH platform can be used to support the implementation of COVID-19 preventive measures, but there are challenges that are being faced by the existing WASH platform in middle- and low-income countries. These include the inadequate availability of water services, lack of basic handwashing facilities, and lack of knowledge and awareness of proper hand hygiene practices such as critical moments for handwashing, proper steps of handwashing, adequate time of handwashing, and the benefits of proper handwashing [15, 16]. Globally, it is estimated that 3 billion people do not have access to most basic handwashing facilities at home and about 43% of the health facilities lack hand hygiene at the point of care [16].

As the fighting against the COVID-19 pandemic continues, it is time to promote good hygiene by improving the existing WASH facilities and installing wash facilities in public areas and buildings where they are needed most with a view to promoting behavioral changes in the communities. In order to ensure sustainable behavioral change, efforts should be made by the government to encourage communities to adopt new behaviors; this can be done through mass education, social marketing, and community mobilization. In counties where the COVID-19 already exists, efforts should be made to ensure that all the current WASH interventions are expanded to reach communities where such services are non-existent. Further, we think that there is a need to ensure constant water supply is made available to enable regular and frequent handwashing. The governments, world over, should raise awareness of their communities on the importance of adhering to basic hygiene and sanitation practices regularly and distribute hygienic kits/soap in areas where some communities cannot afford them.

In conclusion, we wish to insist that WASH practices especially HWWS when applied regularly will serve as a barrier for further transmission of COVID-19. We call upon countries worldwide to consider the need to expand investment in WASH services because these will function as an important mechanism in mitigating secondary effects of COVID-19 in communities during the recovery phase. In our opinion, if WASH services are not well invested, it will increase the risk of transmission of COVID-19 as well as outbreaks of other water-related diseases.

*Let us all not forget to adhere to the measures recommended by the WHO; together, we can fight the COVID-19 pandemic.*

## Data Availability

All the data used are from the references provided.

## Abbreviations

COVID-19: Coronavirus disease-2019
HWWS: Handwashing with water and soap
RNA: Ribonucleic acid
SARS-CoV-2: Severe acute respiratory syndrome coronavirus 2
USA: United States of America
WASH: Water, sanitation, and hygiene
WHO: World Health Organization
